# Application of Electrospun Water-Soluble Synthetic Polymers for Multifunctional Air Filters and Face Masks

**DOI:** 10.3390/molecules27248753

**Published:** 2022-12-09

**Authors:** Gerardo Grasso, Daniela Zane, Sabrina Foglia, Roberto Dragone

**Affiliations:** 1 Istituto per lo Studio dei Materiali Nanostrutturati Sede Sapienza, Consiglio Nazionale delle Ricerche, P. le Aldo Moro 5, 00185 Rome, Italy; 2 Istituto dei Materiali per l’Elettronica ed il Magnetismo, Consiglio Nazionale delle Ricerche, Parco Area delle Scienze 37/A, 43124 Parma, Italy

**Keywords:** electrospinning, indoor air, airborne pathogens, face masks, COVID-19, particulate matter

## Abstract

The worsening of air quality is an urgent human health issue of modern society. The outbreak of COVID-19 has made the improvement of air quality even more imperative, both for the general achievement of major health gains and to reduce the critical factors in the transmission of airborne diseases. Thus, the development of solutions for the filtration of airborne pollutants is pivotal. Electrospinning has gained wide attention as an effective fabrication technique for preparing ultrafine fibers which are specifically tailored for air filtration. Nevertheless, the utilization of harmful organic solvents is the major barrier for the large-scale applicability of electrospinning. The use of water-soluble synthetic polymers has attracted increasing attention as a ‘green’ solution in electrospinning. We reported an overview of the last five years of the scientific literature on the use of water-soluble synthetic polymers for the fabrication of multifunctional air filters layers. Most of recent studies have focused on polyvinyl alcohol (PVA). Various modifications of electrospun polymers have been also described. The use of water-soluble synthetic polymers can contribute to the scalability of electrospinning and pave the way to innovative applications. Further studies will be required to fully harness the potentiality of these ‘greener’ electrospinning processes.

## 1. Introduction

The worsening of air quality, especially indoor air is an increasingly urgent human health issue of modern society. People spend most of their daily time in indoor environments: transportation, e.g., subway trains/rail stations, schools, and workplaces. Poor air quality conditions could be associated with a wide range of effects on the exposed populations, from the onset of the symptoms of the so-called *sick building syndrome* [1], which includes the practical implications for people’s work performances and productivity, to the onset of health problems in the general population [2]. The latter could include the onset of respiratory allergies, chronic respiratory diseases, and cardiovascular diseases [3]. Madruga (2022) has recently published a comprehensive review about the indoor air pollution topic [4]. Air pollutants such as particulate matter (PM10 and PM2.5) are widely recognized as significant risk factors for respiratory disease-related morbidity and mortality worldwide [5]. The main anthropogenic sources of PM are industrial activities, combustions (e.g., domestic heating and vehicular emissions) and intensive farming. The chemical composition of PM is complex and shows a high degree of variability, depending on the type of emission sources, as well as on the atmospheric transformations that may occur [6,7,8]. To properly assess the risk factors with regard to human health, the chemical composition of PM should be carefully considered, but the full understanding of the health risks from the exposure to multiple components of PM is still challenging. One of the key component of PM is biological aerosols or bioaerosols [9]. It has been reported that approximately 24% of atmospheric particles and 5–10% of the total suspended particulate mass are composed of bioaerosols [10]. Bioaerosols can be defined as mixtures of airborne viable or non-viable biological particles that can comprise bacteria, fungi, spores, and viruses, released into the atmosphere from natural and anthropogenic sources [11]. In recent years, bioaerosols have attracted increasing attention as a major global health concern, particularly for vulnerable subjects, whose exposure to bioaerosols can lead to the onset of a number of pathologies, from non-allergic irritative reactions to serious infections [12]. To date very few studies have focused on deepening the knowledge of the sources and transport pathways of bioaerosols [13]. For the development of effective control and prevention measures, it is now becoming all the more imperative to find a comprehensive understanding of the aerobiological aspects that may contribute to the transmission of bioaerosol-associated infectious diseases.

Many studies conducted during the COVID-19 pandemic have suggested a presumptive “carrier effect” or “boost effect” of PM on the SARS-CoV-2 spread [14]. Together with PM10 and PM2.5, possible relationships between outdoor air pollutants (mainly O_3_, SO_2_, NO, NO_2_, and CO) and the COVID-19 outbreak have also been extensively reported in the literature. In this framework, the role of ammonia (NH_3_) should also be considered, especially for the effect on pH of atmospheric water and the consequent changes in the net surface charge of the airborne viral particles [15].

All the difficulties and the imposed restrictions generated by the outbreak of the COVID-19 pandemic have made the improvement of indoor air quality an even more urgent goal, both for the general achievement of major health gains and also because poor air quality can be a critical risk factor in the transmission of airborne diseases. The studies dedicated to the characterization of the microbiological and chemical status of the indoor air of several indoor environments are now largely available. These indoor environments include university libraries [16,17], healthcare and care facilities [18,19], heritage and museum buildings [20], places of worship [21,22], underground and train stations [23], and agricultural buildings [24]. Against this background, the development of effective solutions for the control of air quality in indoor environments is becoming more urgent [25]. The recent outbreak of COVID-19 has also highlighted the importance of personal protection devices and ambient air filtration systems with improved performances, in order to address the public health issues with the development of ad hoc technological approaches. It is known how the use of face masks has played a prominent role in controlling the spread of infectious droplets and aerosols, especially in indoor environments, thus containing the spread of the transmission of airborne diseases [26].

**Figure 1:**
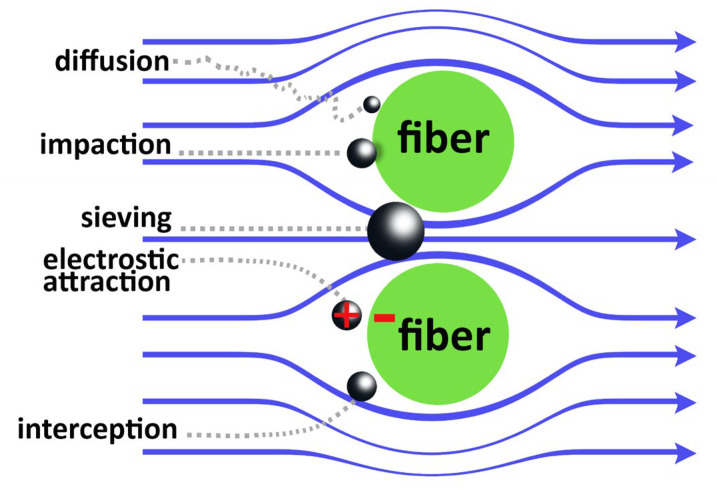
Capture mechanisms involved in air filtration.

The provision of adequate ventilation, as well as the development and implementation of innovative solutions for the filtration, disinfection, and/or adsorption of harmful substances, has recently drawn significant interest for the progress of indoor air purification solutions. Mechanical filtration via polymer fibrous filters is a simple and extensively utilized technique to substantially retain atmospheric particulate matter [27]. With the increasing demand for high-performance filtration which is also dedicated to the removal of other airborne pollutants, including VOCs, inorganic gases, and bioaerosols, considerable research has been conducted to develop and design novel polymeric-based fibrous filters. In addition, modern manufacturing processes should be increasingly more oriented towards more environmentally sustainable processes [28]. Electret air filters are microbus filters widely used in filtration devices due to their cost-effectiveness, high filtration efficiency, and low pressure drop. Examples of electret air filters filter include HEPA filters, N95 and N99 respirator filters, clean room filters, and automotive cabin air filter [29]. In particular, electret melt-blown polypropylene nonwoven filters are predominantly used for respirators and for the manufacturing of the core filter layer of commercial masks [30]. According to the particle sizes, various capture mechanisms are involved, i.e., sieving, inertial impaction, interception, diffusion, and electrostatic attraction (Figure 1). The latter relies on the electrostatic adsorption capacity of a polarized microfibrous membrane.

One of the main drawbacks of electret melt-blown nonwoven filters is the dissipation of the electrostatic charge of the microfibers (e.g., due to use, moisture, or to ethanol disinfection procedures for reuse). That results in a sharp decline in the filtration efficiency of traditional face masks and a reduction in the service life of the electret melt-blown nonwoven filters [31]. Another possible limitation in terms of filtration efficiency is that melt blowing mainly produces microstructured filters [32].

Increasing efforts should be devoted to the development of advanced materials, characterized by improved performances and the sustainability of the manufacturing processes. Electrostatic fiber spinning or ‘‘electrospinning’’ is a well-known technique that has recently gained wide attention as a straightforward and versatile process for the preparation of a variety of continuous fibers of nanoscale diameters with distinctive physical and chemical properties [33,34,35]. Electrospinning ′s capabilities in the fine tuning of nanofiber properties have been harnessed in many applications to surpass the drawbacks encountered with the conventional methods used for the manufacturing of filtering fibers. More specifically, electrospinning is an advanced nanotechnological technique for processing polymeric fibers with nanoscale morphology and a higher surface area, compared to the conventional melt-blown nonwoven filter microfibers. Moreover, the electrospinning process offers opportunities for the fine-tuning of the surface functionality, thus representing a concrete opportunity for the future development and exploitation of electrospun nanofiber membranes. Therefore, electrospinning is a promising solution, with a wide potentiality for the large-scale production of electrospun nanofibers for advanced applications in air purification [36,37,38,39,40].

Recently, the use of water-soluble synthetic polymers has attracted increasing attention as ‘green’ solution for electrospinning [41] and a special issue entitled “Advances in Water-Soluble Polymers” by *Molecules* clearly demonstrates the interest in this topic [42].

In this review, we reported an overview of the scientific literature (in the period from 2017 to 2022) on the use of water-soluble synthetic polymers (i.e., polyvinyl alcohol, polyethylene oxide, polyacrylic acid, and polyvinylpyrrolidone) for the fabrication of nanostructured materials for possible application as multifunctional air filters and filter layers in face masks. We focused on works that covered several key aspects: (i) use of water or low-toxicity solvents (i.e., ethanol and acetic acid) for the preparation of the electrospinning solutions; (ii) the use of physical crosslinking or non-toxic crosslinking agents; (iii) modifications to fabricate multifunctional nanofibers with enhanced properties in terms of microbial and chemical air pollutants removal; and (iv) characterization of filtration performance efficiency and antibacterial activity of electrospun materials.

## 2. Electrospinning: From Basic Principles to the Use of Water-Soluble Polymers for ‘Greener’ Electrospinning Processes

Electrospinning methods have been widely employed for the fabrication of nonwoven materials and advanced fiber-based applications pertaining to air filtration [43]. The basic experimental apparatus and setup for electrospinning includes a metallic needle (spinneret) connected to a syringe, a high-voltage power supply, and a grounded collector. The basic electro-hydrodynamic principle behind the electrospinning process involves the formation of a conical shape droplet emerging from the spinneret, known as the Taylor cone. The application of a high-voltage electric field between the conductive spinneret and the grounded collector leads to the generation of repulsive electrostatic forces on the droplet surface. When the high-voltage electric field attains a critical value, i.e., the electrostatic forces overcome the surface tension and viscosity of the fluid, an electrohydrodynamic jet is ejected and reaches the grounded collector. At this stage, the solvent evaporates and random or aligned fibers are produced [44]. The control over the produced electrospun nanofibers can be obtained by changing different parameters. Indeed, several parameters can affect the characteristics of the electrospun nanofibers (Table S1) and can be classified as (i) solution-related parameters, e.g., the concentration of the polymer melt or solution; (ii) processing parameters, e.g., the applied voltage, solution flow rate, and type of collector; and (iii) environmental parameters, such as ambient temperature and humidity [45]. Haider at al. (2018) provided a comprehensive review on how the electrospinning parameters and the adjustment of the process variables can affect the fabrication of the electrospun nanofibers [46]. Among the processing parameters of electrospinning, the type of collector used can influence the orientation of the fibers and can affect the fiber morphology and the reduction in nanofiber diameters and can improve the mechanical properties of the fiber mat.

**Figure 2:**
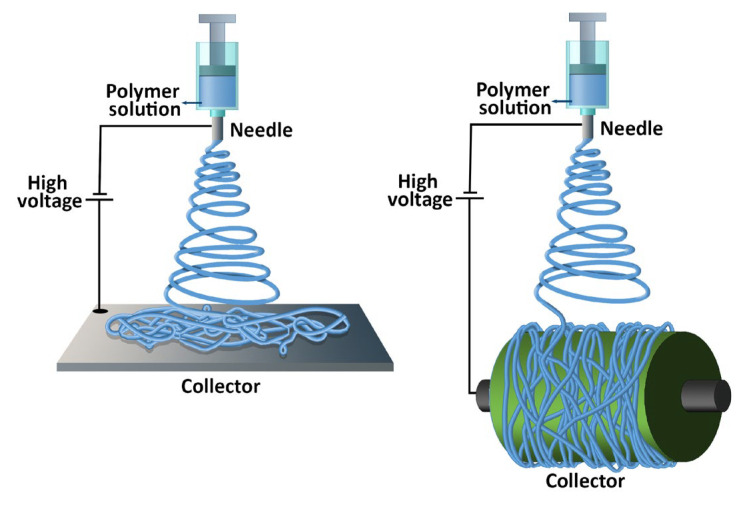
Schematic illustration of electrospinning methods employed for water-soluble polymers for the fabrication of air filters and face masks.

The collector can be, e.g., a stationary plate or a cylindrical rotating drum (Figure 2).

The use of a stationary collector may result in randomly oriented nanofibers, while the use of a rotating drum collector results in parallelly oriented nanofibers. Indeed, the cylindrical rotating drum (also known as a collector drum) is a type of collector used to collect a continuous and uniform alignment of nanofibers, whose rotation leads the fibers to align perpendicularly to the rotation axis. Many recent works have described the use of a rotating drum.

Electrospinning is not limited to synthetic or natural origin polymers but can also be applied to metals, metal oxides, ceramics, and various organic/inorganic composite systems. In some cases, polymers are just used as carriers or sacrificial templates to enhance the spinnability of some precursors, e.g., cyclodextrins [47]. The incorporation of inorganic nanomaterials such as nanoparticles (NPs) into electrospun polymers can be obtained using different methods. The most straightforward method used is the direct dispersion of NPs into the polymeric solution before the electrospinning step [48]. However, this procedure can lead to a limited NP loading capacity and to their poor dispersion in the structure of the resultant composite nanofibers. For synthesizing hybrid organic–inorganic nanofibers, sol-gel processing is a more suitable solution. Another approach is based on the in situ generation of nanoparticles, e.g., by incorporating amounts of precursor into the polymer mixture [49,50].

Despite all the advantages, electrospinning is still poorly applied for the fabrication of nanofiber air filters. The use of harmful and/or flammable organic solvents in electrospinning is the major barrier for the large-scale applicability of electrospinning methods. The main concerns regard the safety, toxicology, and environmental issues related to the use of harmful and/or flammable organic solvents.

Presently, the Chemical Control Regulation in the European Union (Registration, Evaluation, Authorization and Restriction of Chemicals or REACH) restricts the most commonly used solvents for electrospinning. These solvents include halogenated (e.g., chloroform, and trifluoroethanol) and other toxic solvents (e.g., dimethylformamide). In addition to the issues related to occupational solvent exposure concerns, the limits to the implementation of the electrospinning methods based on organic solvents at the industrial level are mainly related to the high costs of the manufacturing equipment, as well as to the extremely cost-intensive proper disposal of the organic solvents [51].

Depending upon the solubility of the materials to be spun, either organic solvents or water can be used as solvents in the electrospinning methods. To spur the scalability of the electrospinning technology in industrial settings, one of the possible solutions relies on the use of water-soluble synthetic polymers.

The water-soluble synthetic polymers described in this review are polyvinyl alcohol (PVA), polyethylene oxide (PEO), polyacrylic acid (PAA), and polyvinylpyrrolidone (PVP). Most of the recent studies reported in the literature described the use of polyvinyl alcohol (PVA) for the fabrication of multifunctional air filters. PVA possesses a semi-crystalline structure commonly harnessed for biomedical applications due to its exceptional hydrophilicity, good adhesiveness, barrier properties, and excellent mechanical properties. Thus, ultrafine PVA fibers may have potential applications in filtration. The wide range of PVA applicability depends on the PVA compatibility with several polymers and natural materials. The hydroxy groups in PVA can provide the possibility of chemical modification either before or after electrospinning. PEO or polyethylene glycol (PEG) is approved by the FDA for clinical use because it is a nontoxic and nonimmunogenic polymer. Electrospun PEO fibers are indeed particularly interesting for biomedical applications due to their excellent biocompatibility. Both PVA and polyethylene oxide (PEO) are frequently reported as being in a comatrix with other materials with poor spinnability to enhance them and then improve their processability [47,52]. Polyvinylpyrrolidone (PVP) is a hygroscopic, amorphous, biocompatible, biodegradable polymer. PVP has been also recognized as safe by the Food and Drug Administration (FDA). In addition, PVP possesses unique physical and chemical features: it is essentially chemically inert, colorless, temperature-resistant, pH-stable, and temperature-resistant. PVP is an excipient extensively used in pharmaceuticals for tablets and capsules and for drug delivery applications. Polyacrylic acid (PAA) exhibits high water absorbance, robust mechanical properties, and known biocompatibility, and it is widely used in various biomedical applications. The hydrophilicity of water-soluble synthetic polymers is a common feature as well as one of the main drawbacks to their application in the filtration of humid gases. Indeed, the phenomena of water adsorbing, swelling, and dissolution can occur. This can seriously affect the morphology and structure of electrospun water-soluble synthetic nanofiber polymers. To address this drawback, a crosslinking step is necessary to provide the post-spinning water resistance and stability of nanofiber membranes, thus preserving the chemical and physical integrity of the electrospun nanofibers when exposed to wet environments. Physical crosslinking methods (thermal or UV-mediated) are frequently reported, as is the use of a non-toxic crosslinking agent such as citric acid. For instance, the physical crosslinking method provides more tunable mechanical properties to the PVA polymer in comparison with other crosslinking methods [53].

## 3. Electrospun Water-Soluble Synthetic Polymers for Multifunctional Air Filtering Applications

To face the challenges of modern air filtration, the characteristics and functionalities of filtering fibers should be adjusted according to the desired applicability. Through treatments during or after electrospinning, it is possible to modify the pristine electrospun nanofibers and make them more performant with respect to the specific application. These modifications may concern the improvement of the mechanical, electrical, and thermal properties, or they may also confer new desirable functionalities, e.g., catalytical and antimicrobial activities. A thermally crosslinked multifunctional polyvinyl alcohol)/polyacrylic acid (PVA-PAA) composite membrane has been reported by [50]. The thermal crosslinking induces esterification between hydroxyl in PVA and carboxyl in PAA, obtaining a PVA-PAA composite membrane with high hydrophobicity. The introduction of superhydrophobic silica nanoparticles (SiO_2_) contributed to the resulting hierarchical structure of the filters, producing a material with high tensile strength (more than 8.5 MPa) and a rough surface. Ag nanoparticles (AgNPs) were formed on fiber surfaces by the post-electrospinning UV-mediated reduction of AgNO_3_**,** to add an antibacterial functionality to the filter. The zone of inhibition test was used to assess antibacterial activity against *Escherichia coli* and *Bacillus subtilis*. The filtration efficiency of the material was also tested using sodium chloride (NaCl) and di-ethyl-hexyl-sebacate (DEHS) particles as model non-biological aerosols, which were representative of inorganic matter [54] and organic matter [55], respectively. The filtration efficiency was expressed as the Quality Factor (Qf), a widely used tradeoff parameter (based on the experimental data of the filtration efficiency and pressure drop):

Qf = −ln(1 − η) ΔP(1)

where η is the aerosol filtration efficiency, ΔP is the pressure drop, and Qf is the quality factor. The Qf values of the PVA-PAA-SiO_2_-Ag NPs nanofibrous membranes (calculated with Equation (1)) were very similar for NaCl and DEHS (0.0339 Pa^−1^ and 0.0384 Pa^−1^, respectively). The filtration efficiency and pressure drop of the PVA-PAA-SiO**_2_** NP membranes were affected by the different levels of SiO**_2_** NPs doping, while extending the duration of the electrospinning process resulted in an increase in filtration efficiency and pressure drop [49]. A similar result was subsequently obtained by the same research group using chitosan (CS) for the fabrication of (CS-PVA) composite nanofibrous membranes. Chitosan is a biocompatible polymer soluble in acetic acid, whose spinnability is improved by PVA. UV irradiation was used as the physical crosslinking method, achieving CS-PVA composite nanofibrous membranes with a good stability in wet conditions below 80 °C. In addition, UV irradiation was used for the post-electrospinning UV-mediated reduction of AgNO_3_ to Ag nanoparticles. The introduction of silica nanoparticles (SiO_2_ NPs) created a hierarchical roughness on the nanofiber surfaces and thus improved the filtration performance and provided self-cleaning features, similarly to that reported in [49]. The CS/PVA@SiO_2_/Ag NPs membranes possess a stable and efficient filtration performance (>98%), which was tested using NaCl and DEHS as non-biological test aerosols (Qf = 0.055 Pa^−1^, Qf = 0.047 Pa^−1^, respectively). A zone of inhibition test on an agar plate was performed to verify the antibacterial activity against *E. coli* and *Bacillus subtilis* [50]. The doping of PVA nanofibers with AgNPs is also described by Blosi et al. (2021). The synthesis of AgNPs was performed using AgNO**_3_** as a precursor and hydroxy-ethyl cellulose (HEC) as a reducing and stabilizing agent (capping agent), forming AgHEC NPs [56]. The AgHEC NPs were also characterized as spherical particles with a hydrodynamic diameter around 60 nm and a positive surface charge (zeta potential ≈ + 4.5 mV). After thermal treatment, the PVA/AgHEC NFs showed an excellent water stability in terms of the maintenance of the shape and porous structure of the nanofilters. The authors have also reported the results of filtration efficiency airflow resistance and antimicrobial activity tests of the filter medium. Such tests were performed under conditions representative for facemasks. Noteworthily, a total bacterial depletion of *Escherichia coli* ATCC 11229 and *Staphylococcus aureus* ATCC 6538 was observed, according to a shake-flask test [57]. The pressure drop and filtration efficiency values were within the range of FFP1 and FFP2 masks, even at the highest filtration velocity of 16.7 cm s^−1^. More specifically, the results of filtration tests performed on 5-layer samples and using nano-aerosolized particles of polystyrene latex and NaCl showed an averaged pressure drop value of 230 Pa and a filtering efficiency towards 97.7% and higher with respect to the requirements of the EN149 standard and a pressure drop in line with FFP1 and FFP2 masks [58].

In addition to AgNPs, other inorganic and organic components have been described for the manufacturing of electrospun water-soluble synthetic polymers with enhanced air filtering performances. Zhang et al. (2019) described the fabrication of a PVA/cellulose nanocrystal (CNC) nanofibrous air filter with high PM removal efficiency and low air resistance. The use of CNCs in the fabrication of electrospun nanofibers has attracted great interest because they originate from renewable resources and possess outstanding mechanical properties. Indeed, Zhang et al. (2019) showed that CNCs acted as nanofillers and mechanical reinforcement agents, both increasing the tensile strength of the PVA fibers and reducing the electrospun fiber diameter. The addition of CNCs also showed to positively improve the filtration characteristics of the fabricated filters. Specifically, the authors report (i) a pressure drop reduction from ∼178 Pa (PVA filters) to 15 Pa (PVA/CNCs filters with 20% CNCs) and (ii) the increased removal efficiencies of PM2.5 and PM10 (generated by burning incense), from approximately 56% and 61% to 82% and 83%, respectively. A further increase in the removal of PM (>95%) was observed after the increase in thickness of the electrospun filters. Among the air filters tested with >95% removal efficiency, the Qf values were above 0.052 Pa^−1^ [59]. Geetha et al. (2022) have recently reported the creation of stable zinc oxide nanoparticles/polyvinyl alcohol/polyvinylpyrrolidone (ZnO NPs/PVA/PVP) composite nanofibered films obtained with the electrospinning method by dispersing homogeneously ZnO NPs with a hexagonal structure into a PVA/PVP polymer blend solution. The antimicrobial activity was evaluated using an antibacterial disc diffusion test against common pathogenic bacterial strains of *Escherichia coli*, *Klebsiella pneumoniae*, and *Streptococcus aeruginosa*. The highest inhibition ability was recorded for *E. coli*. for all the ZnO NP concentrations tested [60]. Borojeni et al. (2021) investigated the effect of a nanoclay addition to a PVP electrospun solution used to fabricate polyvinylpyrrolidone (PVP) electrospun layers. The addition of nanoclay to electrospun polymers has been reported to modify the crystallinity and alignment of the polymer chains. In addition to these, the addition of nanoclay exerted several effects: (i) an increase in fiber roughness; (ii) a decrease in pore size; and (iii) an increase the range of the fiber size distribution. The latter effect has been related to the increase in solution conductivity (from 1.7 ± 0.05 µS/cm to 62.7 ± 0.19 µS/cm) produced by the increased wt% nanoclay (from 0 wt% to 25 wt%), the subsequent destabilization of the electrospun jet, and an increase in the range of the fiber size distribution. No crosslinking method has been reported, and the authors suggested that further research will be required to investigate the influence of the possible crosslinking methods and the coaxial electrospinning strategies on improving the water resistance as well as the aerosol filtering performance of these electrospun PVP–nanoclay composites. However, the PVP–nanoclay composites were characterized by a combination of fine and thick, rough fibers that makes them attractive for air-filter applications [61]. Ayodeji et al. (2022) have recently explored the development of a PVA/ZnO electrospun nanoweb functional surface to be used as a component of a multilayer barrier of functional face masks. Future studies will be required to characterize the PVA/ZnO nanowebs, with the assessment of the antiviral and/or antibacterial properties [62]. Sol-gel electrospinning processing has been proposed by des Ligneris et al. (2019) for the engineering of composite nanofiber membranes of PVA crosslinked with copper (II) acetate. The use of sol-gel electrospinning allowed the incorporation of copper (II) as a sol within a polymer matrix, leading to a homogeneous distribution on the fiber surface. In this proposed polymer–metal acetate crosslinking method (polymer to metal ratio of 60:40 wt%), acetic acid was used as a stabilizer to avoid the hydrolysis of the PVA chains. Using this crosslinking method, the PVA/copper nanofiber membranes showed a moisture-resistance behavior, with no degradation or swelling, and limited membrane fouling. The air filtration efficiency performance was assessed against 0.3, 0.5, 0.7, 1, 2, and 5 μm KCl crystals, and it was in the range of 99.71–99.99%. The assessment of antibacterial activity against *Escherichia coli* was also carried out via the agar diffusion plate test, following a qualitative test procedure adapted from the standard ISO 20645 protocol for the determination of antibacterial activity for textile fabrics [63,64].

The PM of subway stations is composed of a significant amount of metal oxide dust, mainly generated by the brakes, the wheel–rail interface, and the vaporization of the metal after the creation of sparks. In particular, the PM in subway stations can be composed of up to 67% iron oxide in PM2.5 [65] and up to 50% in PM10 [66]. An interesting Fe_3_O_4_ MNP-decorated PVP nanofiber filter was proposed by Kim et al. (2017), with possible applications for the removal of airborne metal oxide dust. Indeed, thanks to the increased magnetic flux density, such a magnetic force-based air filtration system enabled the filter to efficiently attract the metal oxide dust. The filtering performance was tested using Fe_2_O_3_ nanoparticles as test metal oxide dust, resulting in a particle collection efficiency of 97% and a pressure drop of about 17 Pa, i.e., without inducing the classical tradeoff between collection efficiency and the pressure drop of the filter [67]. Various Cu/Zn ratios were tested by [68] for the fabrication of CuZn polyvinylpyrrolidone (CZ-PVP) nanofibers. The PVP was used as a polymer to control the viscosity of the precursor solution. The antibacterial effects of CZ-PVP nanofibers against *E. coli* were assessed using the plate count method. The percentage of antibacterial efficiency E(%) was calculated by using the following equation:

E(%) = (A − B/A) × 100(2)

where A is the number of bacteria colonies grown from the average of 0 h of contact time, and B is the number of bacteria colonies grown from each CZ nanofiber. The CZ-PVP nanofiber calcinated at 353 K exhibited antibacterial effects quicker (1 hr E% = 100%) than the CZ-PVP nanofiber calcinated at 873 K. Zhang et al. (2020) developed another example of a multilayer composite membrane system with antibacterial properties. The system was composed of a N-halamine nanofibrous bipolymer PVA/P(ADMH-NVF) as a middle layer with enhanced mechanical stability and antibacterial property, within two external layers made of a polyvinyl alcohol/chitosan electrospun membrane (PVA/CS). The use of PVA as polymer matrix increased the poor spinnability and reduced the intrinsic mechanical brittleness of CS; N-halamine could be electrospun alone, but the use of PVA enhanced the mechanical property of the resulting fibers, with a tensile strength of 6.1 MPa. N-halamines are heterocyclic organic compounds and biocides active for a broad spectrum of bacteria, fungi, and viruses [69]. Indeed, the disc diffusion method confirmed the antibacterial activity against *E. coli* (ATCC: 8739) and *S. aureus* (ATCC: 29213). In addition, high filtration efficiencies of 99.3% for NaCl and 99.4% for a DEHS aerosol test have been reported, while holding a relatively low pressure drop of 183 Pa for NaCl and 238 Pa for the DEHS particles. Unlike other works reported in this review, glutaraldehyde was used as PVA crosslinking agent and a chlorination step using sodium hypochlorite [70].

Lv et al. (2019) [71] described the fabrication of PVA and konjac glucomannan (KGM)-based nanofiber membranes, loaded with ZnO nanoparticles (ZnO@PVA/KGM) for air filtration. Konjac glucomannan is a polysaccharide extracted from tubers of the devil′s tongue plant (*Amorphallus rivier*) with good film-forming ability, as well as bioactivity. Electrospun fibers were crosslinked through thermal crosslinking by esterification between the hydroxyl groups of PVA and KGM and the carboxyl groups of CA. Thermal crosslinking led to a significant improvement in both the water resistance and the mechanical properties of the ZnO@PVA/KGM membranes. ZnO NPs (30 ± 10 nm) were encapsulated by the nanofibers (diameter > 100 nm), and when the ZnO concentration increased from 1.0 to 2.0 wt%, the filtration efficiency was affected (it decreased to 98.01% with a pressure drop increase to 158 Pa), perhaps because the high ZnO concentration decreased the spinnability of the precursor solution. The filtration efficiency of the ZnO@PVA/KGM membrane (1.0 wt% ZnO) was tested for 30 cycles and 300 min. The results showed that the ZnO@PVA/KGM membrane maintained more than 97% filtration efficiency even after 150 min and could be reused for as many as 30 cycles, with the filtration efficiency maintained at more than 98% for 0.3 μm particles (100% for 5 and 10 μm particles). Such a fibrous membrane showed photocatalytic properties (tested using methyl orange) and antibacterial activity against *Escherichia coli* and *Bacillus subtilis*. The resistance of PVA/KGM nanofiber membranes to water was also tested by evaluating the weight loss of W(%) upon water immersion and was calculated as follows:

W(%) = [(M_1_ − M_2_)/M_1_] × 100 (3)

Such tests have highlighted the effect of CA concentration on the improvement in the resistance to water. Indeed, the weight loss of the membranes was significantly prevented when the CA concentration was 0.6 wt%. The membranes with lower amounts of CA became severely swollen and deformed when immersed in water.

In addition to konjac glucomannan, in the recent literature many other examples of compounds of natural origin have been reported for the functionalization or enhancement of the characteristics of electrospun water-soluble synthetic polymers. PVP has been used by Lu et al. (2021) to prepare shellac–PVP electrospun air filter membranes. Shellac is a resinous product obtained from the secretion of the female “lac bug” (*Kerria lacca*) living on trees in Southeast Asian countries and recognized as safe by the FDA. To overcome the poor mechanical properties and bad light transmittance of pristine shellac fiber, a small amount of PVP was added into the shellac solution. The tests performed showed filtration efficiencies as high as 95% and 98% for PM2.5 and PM10, respectively. The pressure drop of 101 Pa was comparable to that obtained using polypropylene nanofibers from commercial surgical masks (76 Pa) [72]. Li et al. (2017) produced keratin/polyethylene oxide (PEO) nanofibers for air filtration by the electrospinning of the blend aqueous solution. The spinning solution was prepared in combination with the high-viscosity polymer PEO because low-viscosity keratin is not suitable for spinning alone. The PEO was then removed using chloroform. It was reported that both the morphological structures and the diameter distributions of the keratin/PEO nanofibers were affected by the content of keratin. The filtration efficiency of the nanofiber nonwoven composites was significantly higher than that of the polypropylene nonwoven fabric used as a control, and it increased with the prolongation of the electrospinning time [73]. Another approach using a minimal amount of PEO as a carrier/sacrificial polymeric matrix was described by Celebioglu and Uyar (2017) to obtain γ-CD electrospun nanofibers for the electrospinning of cyclodextrins (CDs). Indeed, without the sacrificial polymeric matrix, the electrospinning of CD solutions in dimethyl sulfoxide/water mixture is challenging compared to the electrospinning of polymeric solutions. This is mainly due to the aggregation of CD molecules in high concentrated solutions that affect the viscosity of the precursor solution. As described by [73], PEO was removed by washing the nanofibers in chloroform, obtaining uniform and bead-free electrospun nanofibers. The capability/capacity of the cage-type crystalline structure of γ-CD short-nanofibers for removal of volatile organic compounds (VOCs) was tested using aniline vapor in a desiccator, i.e., no flow tests have been performed. Aniline, a toxic aromatic amine, is a well-known highly toxic and carcinogenic VOC [47]. Cui et al. (2021a) recently investigated the PM1.0 removal ability of a PVA–tannic acid composite nanofiber membrane filter, using a thermal crosslinking method. Tannic acid is a naturally derived polyphenol compound extracted from plants and microorganisms; it has multiple phenolic hydroxyl groups on each branch, which can be used as hydrogen bonding sites for linear polymers of PVA. For a comprehensive review of the applications of tannic acid in membrane technologies, see [52]. The results from mechanical tests pointed out the increase in mechanical strength from 7.9 MPa to 12.4 MPa, and the elongation increased from 31% to 78%, compared to pure PVA nanofiber membrane. Air filtration tests (using DEHS and NaCl aerosols) showed that the PVA-TA filter can maintain high filtration efficiency (99.43%) and a low pressure drop (35.5 Pa) after ten cycles, with a Qf of 0.15 Pa^−1^. Such a PVA-TA nanofiber membrane was also tested as the core filter layer in a protective mask. The filtration stability of these masks was explored through 20 consecutive filtration tests [74]. Another recent publication from the same authors described a flexible and transparent thermal crosslinked (PVA)-sodium lignosulfonate (LS) nanofiber membrane, which was prepared by Cui et al. (2021b) and tested for PM2.5 removal. High PM2.5 filtration efficiency (99.38 ± 0.03%) and a low pressure drop (25.5 ± 0.5 Pa) were observed after 10 continuous cycles. Sodium lignosulfonate is used in the food industry as a de-foaming agent for paper production and in adhesives for food-contact materials. Even though LS possesses preservative properties, the antibacterial activity of the PVA-LS nanofiber has not been tested [75]. The fabrication of a novel poly-(vinyl) alcohol (PVA)/carbon nanoparticle (CNP)/tea leaf extract (TLE) a functionalized nanofibrous air filter (FNA), was recently reported by Senthil et al. (2022). The use of CNPs has enhanced the mechanical properties of the air filter membrane, providing a reinforcement effect with a combination of tensile strength and flexibility. Compared to PVA:CNPs and PVA filters, the results from filtration tests showed the excellent PM filtration properties of PVA:CNPs:TLE nanofilters (PM2.5 and PM10–2.5 with removal efficiencies of 99.25% and 99.29%, respectively), with a pressure drop of 110 Pa. Antimicrobial activity was tested against *Staphylococcus aureus* CECT240 (ATCC 6538p) and *Escherichia coli* ECT434 (ATCC 25922) strains using the disc diffusion method. The combination of CNPs and TLE with PVA-based FNA improved their antibacterial activity as well as the metal adsorption of airborne particles. Water absorption and desorption significantly (*p* < 0.05) increased in PVA: CNPs: TLE. The authors suggested that this could be attributed to the hydrophilic nature of the tea leaf extract and the formation of a hydrogen bond between TLE and PVA. Therefore, a durability test of FNA has been performed, revealing a high durability of FNA after 10 hrs [76]. Jaski et al. (2022) have recently reported the fabrication of a zein-based nanofiber material using two kinds of PVA with the alcoholysis degree of 98–99% and 87–89% as auxiliary spinning polymers. Zein is a hydrophobic prolamine-rich protein extracted from corn (*Zea mais*) which is now strongly emerging as new material for a number of applications in biomedical/pharmaceutical, textile, food packaging, and environmental fields. Regarding the latter application, zein’s ability to remediate air pollutants such as PM, formaldehyde, and carbon monoxide have been previously reported [77]. In Jaski et al. (2022), it has been reported that the nanofiber diameter decreases gradually as the zein content increases. The alcoholysis degree of PVA affected both the air filtration efficiency and the micromorphology of nanofibers, i.e., the low-alcoholysis-degree PVA exhibited better micromorphology and filtration efficiency. More specifically, the latter ranged between 99.32 and 99.99% (for larger 10 µm particles), 99.32% and 99.82% (for 2.5 µm particles), and 99.01% and 99.88% (for 0.3 µm particles). Therefore, through the control of the zein content and proper selection of the PVA alcoholysis degree, the filtration efficiency of the nanofibers, as well as the micromorphology of the nanofibers, can be appropriately tuned. Concerning the surface hydrophobicity and wettability of the materials, with the increase in zein the contact angle of the nanofibers increased, indicating the improved hydrophobic properties, and the nanofabric with high-alcoholic-degree PVA possessed a higher contact angle than that with the low alcoholic degree [78].

Li et al. (2018) described a composite nanofiber filter using PVA and silk fibroin. Silk fibroin is a protein harvested from spiders and worms (*Nephila clavipes* dragline and *Bombyx mori*), with excellent mechanical properties, good biocompatibility, and slow biodegradability [79]. The PM2.5 filtration efficiency is calculated as follows:

H = (C1 – C2)/C1(4)

where C1 and C2 (μg/m^3^) are the mass concentrations of PM between the two sides of the filter. After 24 h of operation, the PM2.5 filtration efficiency remained at 98.97% [80].

Interestingly, Kim et al. (2021) [81] have described the use of citric acid as a crosslinking agent for a PVA nanofibrous composite nonwoven. Citric acid (CA) is a biocompatible, water soluble, non-toxic, and inexpensive tricarboxylic acid. Increasing the amount of CA had significant effects on both the water stability of the PVA nonwovens and the resistance to humidity. The water stability of PVA nonwovens was evaluated by measuring the gel fraction.

Gel fraction (%) = Ws/Wi × 100(5)

where Wi and Ws are the dry weight of the PVA nonwovens before and after immersing in water, respectively. Chemical crosslinking due to CA contributed to the increased gelation parts of the PVA nanofibers. The particle filtration efficiency and the airflow resistance of the composite PVA nonwovens after humidification were much lower compared to the ones before humidification. With the increase in CA content, the reduction in PFE by humidification was decreased. It has been reported that the crosslinking conditions did not significantly change the average fiber diameter pore sizes and filtration efficiencies. Similarly, De Oliveira et al. (2021) [82] reported the use of citric acid as a non-toxic and low-cost crosslinking agent in electrospun PVA. The surfactant Triton X-100 has been used to enhance the manufacturing of nanofibers by decreasing the nanofiber diameter, as well as by reducing the formation of beads. The results showed that the concentration of Triton-X affected different characteristics: median fiber size, filter porosity, thickness of the filter medium, and the Darcian permeability constant. A satisfactory humidity resistance of the filter media with 0.25 wt% Triton X-100 showed a maximum 4.1% change in the pressure drop after exposure to 90% RH for 60 min. The morphology of the nanofibers can affect the air filtration performance of the membrane. Indeed, membranes with a uniform stick-like fiber structure can be characterized by a reduced efficiency in PM filtration, and possible disadvantages in terms of pressure drop and air permeability can result [83]. The fabrication of a multi-hierarchical structure curved-ribbon nanofiber membrane was recently reported by Deng et al. (2022). Electrospun PVA was used as a nanofiber matrix and sodium sulphobutylether-β-cyclodextrin (SBE-βCD) for improving the efficiency of the capturing of PM, harnessing the high-polarity sulfobutyl functional groups. After substituting the core-filter layer of the commercial medical mask, the filtration efficiency of the assembled mask was 99.9% for PM2.5 and PM10, with a low pressure drop (59.5 Pa) and a long-term high-performance filtration (20 cycles) [84]. 

The modifications of water-soluble synthetic polymers and corresponding properties are resumed in Table 1.

## 4. Focused Discussion on Critical Issues

One of the main challenges facing the industrial scaling-up of the electrospinning processes is the reduction in the use of harmful and/or flammable organic solvents. When it comes to mass productivity, the environmental impacts as well as the human health consequences must be carefully considered. The use of ‘green’ solvent in the fabrication of electrospun polymeric membrane is considered one of the key aspects that could overcome such obstacles. Water-soluble synthetic polymers contain hydrophilic functional groups that make them soluble in water and many non-aqueous solvents with lower toxicity compared, e.g., to halogenated solvents, are frequently used for electrospinning processes.

**Figure 3:**
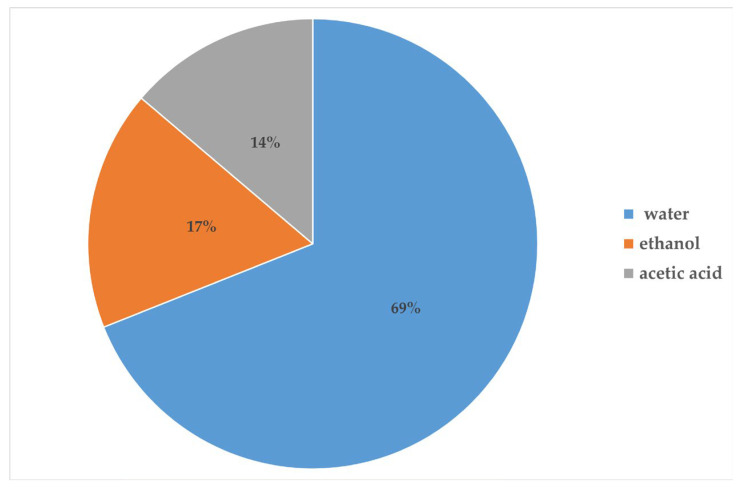
Pie chart of solvents used for electrospinning of water-soluble synthetic polymers de-scribed in this review.

Concerning the works described in this review, Figure 3 shows that water (which is meant to be ultrapure, distilled, or deionized water) is the most frequent solvent used for the electrospinning of water-soluble synthetic polymers (almost 70%), followed by ethanol (17%), and acetic acid (14%). Very few works described the use of harmful solvents, such as glutaraldehyde (as a crosslinking agent), sodium hypochlorite (for the chlorination step) [70], and chloroform, to remove PEO when used as a sacrificial polymer [47,73]. Some works have reported the use of ‘green’ crosslinking methods, such as thermal crosslinking [49,58,71,74,75], UV curing [50], polymer–metal acetate crosslinking [64], and the use of citric acid as a crosslinking agent [81,82]. Most of the articles reported in this review did not describe any crosslinking step, and they did not report any water stability tests. The polymer crosslinking is a crucial post-spinning step to face the intrinsic hydrophilicity of water-soluble synthetic polymers, providing the suitable chemical and physical stability of nanofiber membranes against water. Indeed, phenomena of water adsorbing, swelling, morphological changes, and, lastly, dissolution can occur. Therefore, the loss of functionality of water-soluble synthetic polymer-based filters when exposed over time to wet environments is the current main limitation of the uses in the filtration of humid gases (ambient air and/or exhaled breath). It is known that the relative humidity of outdoor and indoor environments can vary greatly and be affected by specific climate and indoor microenvironmental conditions. The relative humidity in exhaled breath was also found to vary depending on environmental and subject factors [85]. In addition, air humidity was found to significantly influence the aerosol filtration performance in terms of changes in filtration efficiency and pressure drop, depending on the hygroscopicity and size of the aerosol particles and the hydrophilicity of the filter media [86]. Finally, as already stated in the introduction, moisture is also a critical factor affecting the duration of electret melt-blown nonwoven filters.

**Figure 4:**
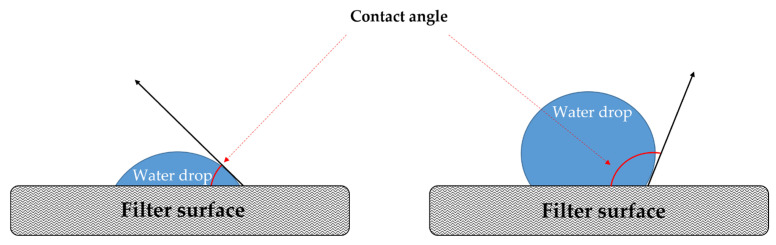
Schematic representation of a water drop on a filter surface.

That said, relative humidity can seriously affect the service life of filters and limits the possible future applications of electrospun water-soluble synthetic polymer-based fibers in indoor air purifier systems and personal air filtration systems (face masks). As said, water stability tests have been performed in every work that described a crosslinking step [49,50,58,64,71,81,82] but two [74,75], or they have been indicated as an object of future investigations [61]. The two works that have not reported a crosslinking step measured the contact angle or wetting angle [72,78]. The contact angle gives an indication of the wettability of the material and the hydrophobicity of the surface (Figure 4). If the contact angle is below 90 degrees, the surface is defined as hydrophilic (‘good wettability’). If the contact angle is above 90 degrees, the surface is defined as hydrophobic (‘poor wetting’). The presence and accumulation of a high amount of moisture on hydrophilic filters can facilitate microbial growth with the decrease in filter efficiency and its deterioration and can also lead to a possible release of microorganisms.

Lu et al. (2021) suggested that one of the reasons for the high filtration efficiency of shellac–PVP electrospun air filter membranes could be ascribable to the good wettability of the nanofiber membrane, with a contact angle of 13 degrees, which is right between the contact angles measured for PVP and shellac of 10.7 degrees and 22 degrees, respectively [72]. This statement seems to not consider the drawbacks that may occur over time for hydrophilic fibers in wet conditions. Li et al. (2020) assessed the effect of zein protein content on the contact angle of nanofibers using PVA with different alcoholysis degrees. It was observed that the contact angle of the nanofibers increased with the increase in the zein content. In addition, the PVA with a high alcoholysis degree (98–99%) possesses better hydrophobic properties than the PVA with a low alcoholysis degree (87–89%) [78].

For all the works reported in this review, filtration efficiency has been measured, and performances have been compared with FFP1 and FFP2 face masks [58] or with conventional filtration materials [78]. Other key parameters such as pressure drop resulted satisfactorily for air filtering applications. For air filters, a pressure drop can be defined as the difference in pressure between the outer and inner sides of a filter. The tradeoff between pollutant collection, removal efficiency, and the pressure drop of the filter must always be considered, especially for the fabrication of face masks. For electrospun nanoscale fibers, if the fibers are densely stacked, the filter efficiency of the small pores is higher, but relatively fast blockage inevitably occurs. In addition, if the pressure drop is high, breathing through the face mask is inevitably reduced and uncomfortable. Thus, for an increased breathability and a more comfortable use of face masks, a low pressure drop in face masks is desired. Satisfactory filtration efficiencies after many cycles of use have also been measured [71,74].

The recent outbreak of COVID-19 has dramatically highlighted the need for personal protection devices against bioaerosols and airborne pathogens. Several works have been dedicated to the fabrication of electrospun nanofiber filters and the relative assessment of antibacterial properties [49,50,58,60,64,70], or they plan to assess them in future studies [62]. All the antimicrobial activity tests have been performed using the zone of inhibition test, basically following ISO 20645:2004 or the protocols adapted from this standard ISO for the determination of the effect of antibacterial treatments applied to woven, knitted, and other flat textiles [63]. To the best of our knowledge, no studies conducted in the last five years on water-soluble synthetic polymer-based filters have reported the assessment of antimicrobial activity by measuring the bacterial filtration efficiency (BFE), which is one of the performances required for medical face mask materials, in agreement with European Standard EN 14683:2019.

Noteworthily, to the best of our knowledge no assessment of antiviral activity of water-soluble synthetic polymer-based filters has been reported in the last five years.

**Figure 5:**
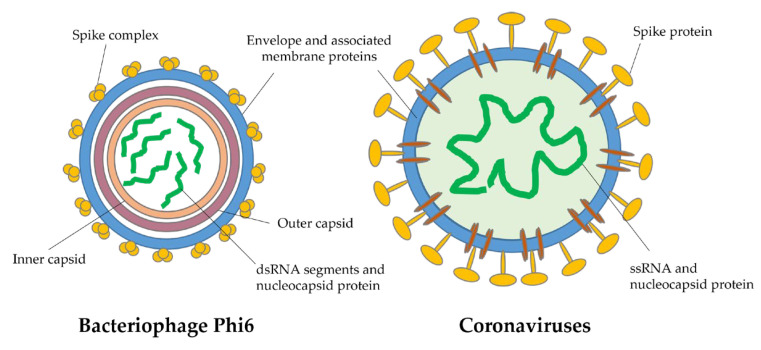
Schematic structures of viral particles of coronaviruses and bacteriophage Phi 6. The latter is considered a good and safe surrogate for studying enveloped RNA viruses.

The viral filtration efficiency (VFE) test is like the BFE test, but it uses bacteriophage phiX174 particles. The bacteriophage phiX174 is a single-stranded DNA (ssDNA) virus that infects *Escherichia coli* bacterium. In future studies, the evaluation of the antiviral performances of filters should be implemented. Bacteriophages are considered good models for the study of airborne viruses because they are safe to use and possess structural features like those of human RNA enveloped viruses such as the coronaviruses and influenza. In particular, bacteriophage Phi6 (Figure 5), a double-stranded RNA (dsRNA) virus that infects the plant pathogen *Pseudomonas syringae* bacterium, has previously been used and is suggested as a good and safe viral surrogate for this kind of study [87,88,89,90,91]. For a more complete characterization of the antimicrobial efficiency of water-soluble synthetic polymer-based filters, the use of nonpathogenic viral surrogates in bioaerosol filtering tests should be further encouraged.

## 5. Conclusions

Several studies have shown how poor air quality favors the spread of airborne dis-eases. A possible defense is represented by face masks that hinder the spread of pathogenic microorganisms, as well as improved ambient air filtration systems. Thus, the development and implementation of innovative solutions for filtration, disinfection, and/or adsorption of the airborne substances harmful to human health is imperative. The effectiveness of the protection depends on the chemical–physical properties of the masks. In this context, electrospun water-soluble synthetic polymers have proved to be a promising and effective solution for multifunctional air filters and face masks for the prevention of infections by pathogenic microorganisms, with a major role in reducing the risk of the outbreak of pandemics.

The use of water-soluble synthetic polymers in the electrospinning processes has the potential to spur the scalability of electrospinning technology in industrial settings and to pave the way for innovative applications in the air filtration field. 

Further studies will be required to fully harness the potentiality of water-soluble syn-thetic polymers for ‘greener’ electrospinning methods. To the best of our knowledge, no studies conducted on water-soluble synthetic polymers reported a performance assessment of antibacterial and/or antiviral activity using bioaerosol tests. These aspects should be carefully considered in future studies.

## Figures and Tables

**Table 1:** Electrospun water-soluble synthetic polymers.

Abbreviation	Chemical Formula	Modification	New or Enhanced Properties	Reference
PEO	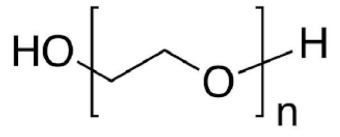	γ-cyclodextrins	Removal of aniline as VOC test compound (no flow tests)	[47]
PAA, PVA	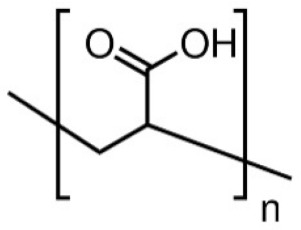 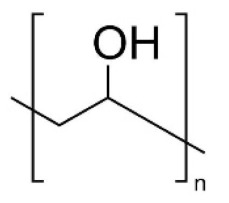	SiO_2_; Ag NPs	Generation of hierarchically structured nanofibers with a rough surface; Filtration of PM2.5 using NaCl and DEHS model aerosols; Antibacterial activity against *E. coli* and *B. subtilis*	[49] ^1^
PVA	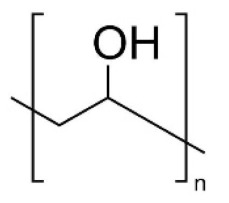	Chitosan; SiO_2_; Ag NPs	Generation of hierarchically structured nanofibers with a rough surface; Filtration of PM10 from NaCl and DEHS model aerosols; Antibacterial activity against *E. coli* and *B. subtilis*	[50]
		AgHCE NPs	Filtration of nano-aerosolized particles of polystyrene latex and NaCl; Total bacterial depletion of *Escherichia coli* ATCC 11229 and *Staphylococcus aureus* ATCC 6538	[58]
		CNTs	Mechanical reinforcement; Filtration of PM2.5 and PM10	[59]
PVA, PVP	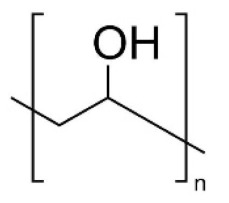 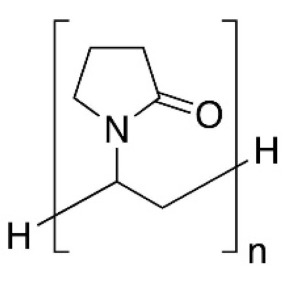	ZnO NPs	Antibacterial activity against *Escherichia coli*, *Klebsiella pneumoniae*, and *Streptococcus aeruginosa*	[60] ^2^
PVA	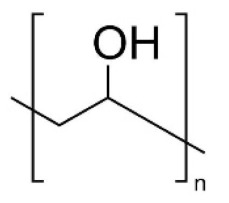	Zn acetate nanowebs	No antimicrobial activity was assessed	[62]
		Copper (II)	Filtration of PM2.5 from KCl aerosol; Antibacterial activity against *Escherichia coli*	[64]
PVP	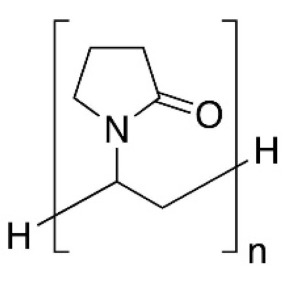	Fe_3_O_4_ magnetic NPs	Filtration of airborne metal oxide dust	[67]
		Cu/Zn	Antibacterial activity against *E. coli*	[68]
PVA	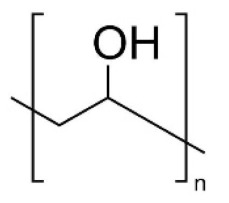	N-halamine; Chitosan	Filtration of NaCl and DEHS model aerosols; Antibacterial activity against *E. coli* (ATCC: 8739) and *S. aureus* (ATCC: 29213).	[70]
		Konjac glucomannan; ZnO NPs	Filtration of NaCl and DEHS model aerosols; Photocatalytic properties (degradation of methyl orange); Antibacterial activity against *Escherichia coli* and *Bacillus subtilis*	[71]
		Shellac	Filtration of PM2.5 and PM10 (generated by burning incense)	[72]
		Tannic acid	Increase in mechanical strength; Filtration of NaCl and DEHS model aerosols (PM1.0)	[74]
		Carbon NPs (CNPs); Tea leaf extract	Reinforcement effect of CNPs; Filtration of PM2.5 and PM10–2.5 (from cigarette smoke); Antimicrobial activity against *E. coli* and *S. aureus*	[75]
		Zein	Filtration of PM2.5 and PM10 (generated by burning incense)	[78]
		Silk fibroin	Filtration PM2.5 and PM10 (generated by burning incense)	[80]
		Sodium sulphobutylether-β-cyclodextrin	Generation of multi-hierarchical structure membranes; Filtration of PM2.5 and PM10 using NaCl and dioctyl maleate (DOM) model aerogels	[84]
		Sodium lignosulfonate	Filtration of PM2.5 from NaCl and DEHS model aerosols	[85]

^1^ PVA-PAA composite polymer. ^2^ PVA-PVP composite polymer.

## Data Availability

Not applicable.

## Supplementary Materials

The following supporting information can be downloaded at: https://www.mdpi.com/article/10.3390/molecules27248753/s1, Table S1: electrospinning parameters for the preparation of electrospun nanofibers described in this review.
