# Supplementary material for: Application of Electrospun Water-Soluble Synthetic Polymers for Multifunctional Air Filters and Face Masks
Source: Molecules. 2022 Dec 9;27(24):8753. doi: 10.3390/molecules27248753 (PMC9784125; doi:10.3390/molecules27248753)
Supplement: Supplementary file 1 [file molecules-27-08753-s001.zip › molecules-2035702-supplementary.pdf]

Table S1. Electrospinning parameters for the preparation of electrospun nanofibers described in this review.

Polymers	Solution-related parameters	Processing parameters	Environmental parameters	Reference
	4 wt% PVA-PAA-SiO2 0.3 wt% AgNO3	Operating voltage: 17 kV; tip-to-collector distance: 12 cm; Solution flow rate: 0.66 mL/h; Rotating collecting drum	T: 25 °C RH: 45%	[49]
	4 wt% PVA-PAA-SiO2 3 wt% CS 0.3 wt% AgNO3	Operating voltage: 20 kV; tip-to-collector distance: 15 cm; Solution flow rate:1.0 mL/h ; Rotating collecting drum	T: 25 ± 3 °C RH: 45 ± 5%	[50]
	15 wt% PVA 0.1 wt% colloidal AgHEC NPs (volume ratio 1:1).	Operating voltage: 30 kV tip-to-collector distance: 20 cm Solution flow rate: 0.02 ml/min	T: 22 ± 2°C RH: 35 ± 5%	[58]
	7 wt % PVA 5%, 10%, 15%, 20% (w/w) CNC;	Operating voltage: 22 kV; Tip-to-collector distance: 10 cm; Solution flow rate: 0.5 mL/h	Room temperature RH: 50 ± 5%	[59]
PVA	0.08g/mL PVA 0.28 g/ mL PVP 50:50(v/v); 0.1 g, 0.5 g and 1 g of ZnO NPs were added separately	Operating voltage: 15 kV; Tip-to-collector distance: 10 cm; Solution flow rate: 0.001 ml/h	N.A.	[60]
	10 wt% PVP 3, 10 and 25 wt% PVP (nanoclay:PVP 3:97, 10:90, 25:75).	Operating voltage: 15 kV; 21-gauge blunt stainless-steel needle; Tip-to-collector distance: 10 to 20 cm; Solution flow rate of 6 μL/min; Rotating collecting drum	RH: 30 ± 1%	[61]
	10% PVA 0.25 g, 0.50 g, and 0.75 g of ZnAc (w/w); 15% ZnAc	Operating voltage: 18 kV; Solution flow rate: 0.015 mL/min; 23-gauge needle	N.A.	[62]

	PVA:Cu 60:40 (w/w)	Operating voltages: 12, 16, 18 kV<br>Needle Tip-Collector Distance: 18, 18, 20 cm<br>Solution flow rate: 0.6, 0.5 and 0.2 ml/h	N.A.	[64]
	10 wt% PVA<br>2 wt% CS<br>10-50 wt% P(ADMH-NVF)	Operating voltage: 20 kV<br>19 gauge needle tip;<br>Tip-to-collector distance: 20 cm;<br>Solution flow rate: 0.5 mL/h	N.A.	[70]
	PVA<br>konjac glucomannan (KGM) solutions (various ratio)<br>ZnO NPs (0, 0.5, 1.0, 1.5, 2.0 wt %)	Operating voltage: 18 kV;<br>Tip-to-collector distance: 15 cm;<br>Solution flow rate: 0.66 mL/h;<br>Grounded metal roller	T: 25 ± 2 °C<br>RH: 40 ± 2%	[71]
	12 wt% PVA<br>3wt% TA	Operating voltage: 15 kV;<br>22-gauge metal needle;<br>Tip-to-collector distance: 15 cm;<br>Grounded drum covered with non-woven fabric	T: 25 ± 3 °C<br>RH: 30 ± 5%.	[82]
	10% wt PVA<br>5 % wt LS<br>2 ‰ wt CTAB	Operating voltage: 18 kV;<br>Tip-to-collector distance: 15 cm;<br>Solution flow rate: 0.6 mL/h<br>Rotating collecting drum	T: 25 ± 2 °C<br>RH: 35 ± 5%	[75]
	8 %w/w PVA<br>0.1% CNPs<br>1 mL TLE	Operating voltage: 22 kV;<br>Needle (23 gauge);<br>Tip-to-collector distance: 20 cm;<br>Solution flow rate: 0.15 mL/h	Room temperature	[76]
	10 wt% PVA;<br>10 wt% zein;	Operating voltage: 14 kV;<br>Tip-to-collector distance: 12 cm;<br>Solution flow rate: 0.8 mL/h<br>.	N.A.	[78]
	9 w/v% PVA<br>3.5% SF	Operating voltage: 20 kV;<br>Micro-needle (22-gauge);<br>Tip-to-collector distance: 20 cm.<br>Solution flow rate: 0.2 mL/h.;<br>Grounded roll-drum collector	Room temperature	[80]

	10 wt% PVA<br>0, 4, 8, 12 wt% citric acids<br>8 wt% sodium acetate	Operating voltage: 25 kV;<br>25 gauge syringe needle;<br>Tip-to-collector distance: 15 cm<br>Solution flow rate of 0.3 ml/hr<br>.	N.A.	[81]
	13 wt.%PVA<br>0, 0.25, 0.50, 0.75,<br>1.0 wt.%Triton X100;<br>5.0 wt.% citric acid	Operating voltage: 27 kV;<br>Needle 0.60 mm inner diameter;<br>Tip-to-collector distance: 10 cm;<br>Solution flow rate: 0.5 mL/h;<br>Rotating cylinder collector covered by cellulose substrate	T: 25 °C<br>RH: 48%	[82]
	10 wt% PVA<br>(various ratio)Sodium sulphobutylether-β-cyclodextrin (SBE-βCD)<br>1 wt‰ Benzyl dimethyl dodecyl ammonium chloride (DDBAC)	Operating voltage: 20 kV;<br>20 gauge needle;<br>Tip-to-collector distance: 16 cm;<br>Solution flow rate: 0.8 mL/h<br>Drum collector with non-woven fabric	T: 25±5<br>RH: 30±5	[84]
PVP	15 wt % PVP;<br>5, 10, 20 wt % Fe_3O_4 MNPs	Operative voltage: 15 kV<br>Tip-Collector Distance: 10 cm;<br>Solution flow rate: 3 mL/h;<br>Grounded aluminum foil collector;	N.A.	[67]
	0.21 g/mL PVP<br>Zn (mole fraction 0.00,0.17,0.30, 0,50,1.00)	Operative voltage: 1.16 kV/cm<br>21-gauge stainless needle;<br>Solution flow rate: 3 μL/min;<br>Drum collector covered with an aluminum foil and rotated at 100 rpm.	N.A.	[68]
	8% w/w PVP<br>40% w/w shellac PVP solutions.	Operating voltage: 15 kV;<br>Needle Tip-Collector Distance: 13 cm;<br>Solution flow rate: 1.0 mL/h;	T: 25 °C<br>RH: 40–60%	[72]
PEO	8% wt blend solutions of PEO and	Operating voltage: 14 kV;<br>Needle Tip-Collector Distance: 24 cm,<br>Solution flow rate: 0.2 ml/h	N.A.	[73]

	keratin (various ratio)			
	1, 2, 3% (w/v)PEO 20% and 40% (w/v) γ- cyclodextrin (γ -CD)	Operating voltage: 10–15 kV; Tip-to-collector distance 10–15 cm; Solution flow rate: 0.5–1 ml/h Grounded stationary cylindrical metal collector covered by a piece of aluminum foil	T: 22 °C RH: 30%	[47]

N.A. not available
